# The anatomy of safe surgical teams: an interview-based qualitative study among members of surgical teams at tertiary referral hospitals in Norway

**DOI:** 10.1186/s13037-024-00389-w

**Published:** 2024-02-19

**Authors:** Magnhild Vikan, Ellen CT. Deilkås, Berit T. Valeberg, Ann K. Bjørnnes, Vigdis S. Husby, Arvid S. Haugen, Stein O. Danielsen

**Affiliations:** 1 Department of Nursing and Health Promotion, Faculty of Health Sciences, Oslo Metropolitan University, St. Olavs Plass, P.O. Box 4, Oslo, 0130 Norway; 2 Department of Health Services Research, Akershus University Hospital, Lørenskog, Norway; 3 Department of Orthopedic Surgery, Trondheim University Hospital, Trondheim, Norway; 4 Department of Health Sciences Aalesund, Faculty of Medicine and Health Science, Norwegian University of Science and Technology, Aalesund, Norway; 5 Department of Anesthesia and Intensive Care, Haukeland University Hospital, Bergen, Norway

**Keywords:** Patient safety culture, Patient safety, Adverse events, Medical errors, Surgery, Quality improvement

## Abstract

**Background:**

In spite of the global implementation of surgical safety checklists to improve patient safety, patients undergoing surgical procedures remain vulnerable to a high risk of potentially preventable complications and adverse outcomes. The present study was designed to explore the surgical teams’ perceptions of patient safety culture, capture their perceptions of the risk for adverse events, and identify themes of interest for quality improvement within the surgical department.

**Methods:**

This qualitative study had an explorative design with an abductive approach. Individual semi-structured in-depth interviews were conducted between 10/01/23 and 11/05/23. The participants were members of surgical teams (*n* = 17), general and orthopedic surgeons (*n* = 5), anesthesiologists (*n* = 4), nurse anesthetists (*n* = 4) and operating room nurses (*n* = 4). Middle managers recruited purposively from general and orthopedic surgical teams in two tertiary hospitals in Norway, aiming for a maximum variation due to gender, age, and years within the specialty. The data material was analyzed following Braun and Clarke’s method for reflexive thematic analysis to generate patterns of meaning and develop themes and subthemes.

**Results:**

The analysis process resulted in three themes describing the participants’ perceptions of patient safety culture in the surgical context: (1) individual accountability as a safety net, (2) psychological safety as a catalyst for well-being and safe performance in the operating room, and (3) the importance of proactive structures and participation in organizational learning.

**Conclusions:**

This study provided an empirical insight into the culture of patient safety in the surgical context. The study highlighted the importance of supporting the individuals’ competence, building psychological safety in the surgical team, and creating structures and culture promoting a learning organization. Quality improvement projects, including interventions based on these results, may increase patient safety culture and reduce the frequency of adverse events in the surgical context.

**Supplementary Information:**

The online version contains supplementary material available at 10.1186/s13037-024-00389-w.

## Background

Patient safety culture has been a proxy for quality of care during the last two decades and is commonly assessed using questionnaires [1, 2]. The most used and recommended questionnaires are the Safety Attitude Questionnaire and the Hospital Survey on Patient Safety Culture [1–3]. A recent study categorizes patient safety culture dimensions into tangible and intangible themes [2]. Tangible themes, including leadership, teamwork climate, and organizational structures, can be effectively captured by questionnaires [2]. Intangible themes with more underlying cultural dimensions, such as power, trust, psychological safety, ethics, and cohesion, are less easily captured by quantitative methods and could be explored by qualitative studies [1–3].

Patient safety culture correlates with safety performance, healthcare professionals’ well-being, patient outcomes, reporting, and incidence of adverse events [4–10]. Adverse events represent unintentional errors or patient injuries resulting from omission or commission in healthcare delivery [11]. Globally, these events affect an average of 10% of hospitalized patients [12, 13], and up to half of the adverse events are estimated to be preventable [11–13]. A significant proportion of the adverse events are related to the surgical context [11–13], and events tend to be more severe, often necessitating additional treatments [14, 15]. These iatrogenic injuries influence the health of patients, their families, and the involved healthcare professionals, who often experience guilt and self-criticism [16]. Healthcare professionals involved in adverse events often experience adverse emotional and physical reactions [16, 17]. Previous retrospective studies from the surgical context demonstrate that human performance deficiencies caused adverse events more than patient-related, organizational, or technical causes [14, 15]. Prospective studies could provide better insight into cognitive errors and potential structural causes of adverse events [11, 14, 15], and an enhanced understanding of intangible cultural themes may prompt initiatives to reduce the frequency of such events [2].

Qualitative evidence assessing patient safety culture is sparse, and further research should be based on more conceptual and theoretical frameworks [18]. Interprofessional surgical teams treat complex cases of vulnerable patients in a high-technological environment, and the dynamic roles and collaboration [19] and the high frequency of adverse events related to surgery [11] necessitate qualitative studies that broaden the understanding of patient safety culture in the surgical context [1, 2, 10]. The objectives of this study were to explore surgical teams’ perceptions and experiences of patient safety culture, including intangible themes and responses to adverse events, to capture their perceptions of the risk for adverse events, and to identify themes for quality improvement and education within the surgical context.

## Methods

### Study design

This study had an exploratory design with a qualitative abductive approach to improve the understanding of patient safety culture in a surgical context [20–22]. With an abductive approach, the research involves iterative movements between inductive and deductive strategies [21, 22]. We used Braun and Clarke’s recommendations for quality practice in thematic analysis to ensure the reporting of the study [23].

### Participants

The participants were 17 members of surgical teams in two Norwegian tertiary hospitals in the South-Eastern Norway Regional Health Authority. In collaboration with middle managers, we purposively recruited and aimed for maximum variation regarding gender, age, and number of years in the specialty [22]. The included participants worked clinically in orthopedic and general surgical teams in departments operating acute and planned surgery on a 24/7 basis. The participation was based on voluntariness, and healthcare professionals in administrative functions or management were excluded.

### Data collection

MV conducted individual in-person interviews between 10/01/23 and 11/05/23. The participants received a mind map to enhance their reflections and note their unsolicited experiences before the interviews [24] (see Additional file 1). The purpose of the mind map was to increase information richness and reduce recall bias [24]. Half the participants brought the mind map and looked at it during the interviews. The mind map was developed on the main topics in the semi-structured interview guide. The interview guide consisted of open-ended questions, allowing participants the flexibility to share broad and in-depth descriptions of their perceptions and experiences [22]. Despite the open-ended questions, the questions were developed from theory, and detailed descriptions and narratives were searched to provide insight into the underlying dimensions of patient safety culture. The main topics of the interview guide were patient safety, adverse events, and near-miss incidents (see Additional file 2). The interviews were conducted close to the participants’ clinical workplace of the participants’ choice and without disruptions. The interview setting was introduced with information about the project and MV, written informed consent, and questions about demographical characteristics. A digital recorder audiotaped and encrypted the dialogues, each lasting between 35 and 90 min. We recruited participants until the data had information richness according to Malterud’s model for evaluating information power [25].

### Data analysis

The analysis process followed Braun and Clarke’s 6 phases for reflexive thematic analysis [26]. MV wrote field notes and transcribed the interviews verbatim to initiate analytic reflections and data familiarization [27]. Each interview provided 9 to 21 transcribed pages, resulting in 230 pages. The initial coding was inductive, semantic, and descriptive, and each of the 81 codes represented ‘an idea’ presented by the participants. The second coding resulted in 56 revised codes. MV used the software NVivo version 12 for analysis transparency, an overview of characteristics across the heterogenous dataset, and memos on reflexivity and interpretations. MV clustered codes with similar ‘core ideas’ and abstracted them into 6 candidate themes relevant to the study’s objectives. MV and SOD discussed the initial coding, initial themes, interpretations, and conceptual abstraction to generate ‘patterns of meaning/shared ideas’ across the dataset. MV generated the results by an iterative process over 3 months, moving between the data, interpretations, and revisions, inspired by literature and discussions with ECTD, ASH, and SOD. This panel of researchers included a physician, an anesthesia nurse, and an operating room nurse. Interpretations were iteratively and critically discussed, close to the raw data material, and related to a contextual understanding and relevant theory. Braun and Clarke describe their process of developing themes through reflexivity, subjectivity, and sensitivity [26]. The analytical process of reflexive thematic analysis is detailed in a table (Additional File 3).

## Results

**Fig. 1:**
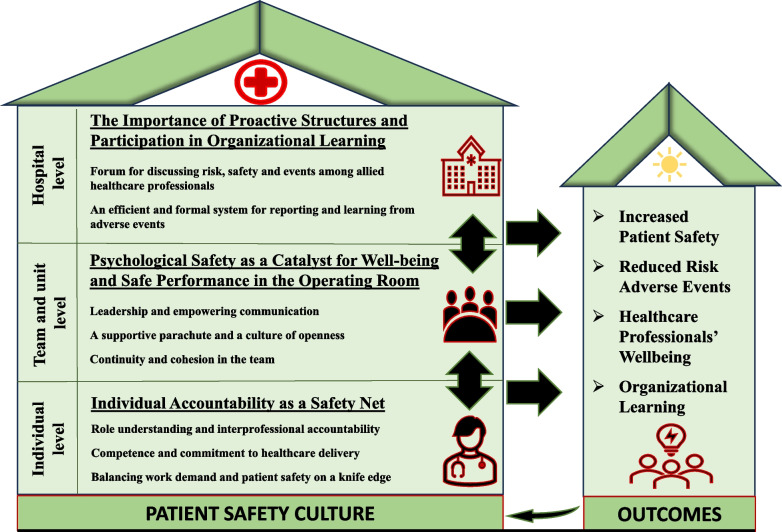
Results. Themes and subthemes: Patient safety culture in surgical teams

Demographic characteristics of the 17 participants are presented in Table 1. The analysis dissected the data on the surgical teams’ perceptions and generated 3 themes and 8 subthemes of shared understandings, as illustrated in Fig. 1. Each participant provided data fitting the themes. The themes are described in the text, and quotations and the number of participants providing data for the subthemes are presented in Table 2.

**Table 1:** Demographic characteristics of the participants in the study

Characteristics	Number (n)	Percentage (%)
Total participants	17	100
**Gender**		
Female	10	59.0
Male	7	41.0
**Age**		
26–40	5	29.5
41–55	9	53.0
56–70	3	17.5
**Profession**		
Physician	9	53.0
Nurse	8	47.0
**Specialty**		
Surgeon	5	29.5
Anesthesiologist	4	23.5
Nurse anesthetists	4	23.5
Operating room nurse	4	23.5
**Years in profession**		
1–5	6	35.0
6–15	4	24.0
≥ 16	4	41.0

**Table 2:** Quotations subthemes (*n *= numbers of participants providing data for the subtheme)

**Theme 1: Individual accountability as a safety net**	
**Subtheme 1a:** Role understanding and interprofessional accountability (*n* = 17)	Operating room nurse (ID02): “I evaluate the situation. Sometimes, they are very focused; other times, we may talk about completely different things as well. So, I have to assess if it is time for me to give that message or wait.” Nurse anesthetists (ID05): “Bringing out the best in each other, being generous with one another…you're probably better at this when you can broaden your perspective, gain some experience, and see others, not just focusing on your tasks.” Anesthesiologist (ID10): “I think it’s important that we help each other and understand each other’s roles so that we can catch if something goes wrong or something isn’t done…that we have an overview of everything, not just ourselves.”
**Subtheme 1b:** Competence and commitment to healthcare delivery (*n* = 17)	Operating room nurse (ID01): “It happens that I read even more carefully and study the pictures more closely to ensure that the surgeon has ordered the right equipment. Is it supposed to be done like this? Occasionally, I make a call, and both orders and everything else turn out differently, and the whole operation also becomes completely different.” Anesthesiologist (ID07): “It’s probably a system designed so that we all act as safety nets, each individual being a safety net. Maybe things go relatively well because everyone is quite thorough, so the safety nets overlap.” Anesthesiologist (ID14): “If you read the newspaper, you can easily think that doctors, especially those who are accused of being arrogant, are, well, I believe that is somewhat misunderstood. I think that one of the things we fear most is not doing well. Yes, we strongly desire to succeed, probably because we are so ambitious, but we also want it to be good for the patients. And how catastrophically it feels personally when you harm a patient.”
**Subtheme 1c:** Balancing work demand and patient safety on a knife edge (*n* = 17)	Operating room nurse (ID01): “Sometimes, the management doesn’t fully understand what we’re dealing with. This is because they have a money bag, and they are terrified of it running dry, while we have a patient, and we are terrified that the patient might die.” Surgeon (ID03): “We are supposed to be trained, which takes more time. You may not be able to do that because you must get through what you need to.” Nurse anesthetists (ID04): “It becomes a situation where you try to be as quick as possible. You often prepare for the next patient while still taking care of the previous one, drawing up medications, and in many situations, that's perfectly fine. If you have a stable patient, you can easily draw up medications in a corner of the room. However, it can become challenging for safety when there is too much pressure to get through things and too much rush.”
**Theme 2. Psychological safety as a facilitator for well-being and safe performance in the operating room**	
**Subtheme 2a:** Leadership and empowering communication (*n* = 17)	Operating room nurse (ID02): “It's much easier to speak up with the younger ones in the operation room. Many, not all, of course, but some have a slightly different attitude towards us as professionals and, generally, teamwork.” Surgeon (ID08): “There are many ways to perform leadership. It is possible to lead with dignity…the leader must make every team member proud of their role in the machinery. Because without this role, it wouldn’t work. Everyone must understand that they are seen and have their position.” Anesthesiologist (ID10): “Lately, I have experienced training from certain personality types, and I can notice that I become insecure, perform worse, lose self-confidence, and fight for my abilities. I believe that how team members talk to each other in stressful and dramatic situations is crucial because I have experienced how it affects me when experiencing scolding and obvious criticism of oneself.”
**Subtheme 2b:** A supportive parachute and a culture of openness (*n* = 17)	Anesthesiologist (ID06): “We must acknowledge that we make mistakes, that it can happen, and that it’s part of the profession. Statistically, things will sometimes go wrong, and we must be able to work with that. We should also ensure that we provide strong support for each other and that everyone is open about the fact that it could be any of us. So, it’s important that everyone feels that we’re all in the same boat.” Surgeon (ID16): “If there had been a culture of fear or ridicule, it would probably have made you feel more nervous. If it sharpens one’s focus, nervousness is good, but being too nervous can be destructive. I feel there’s a low threshold for asking for help and admitting that I’m uncertain: ‘Can you help me?’ Those are issues that probably contribute to providing that freedom to reduce the percentage.” Nurse anesthetists (ID11): “One talks about positive events or like 'oh, what did I just do’, ‘have you heard?' sort of like 'this wasn't good' or supports each other if there are things to share. So, we have a very informal, but it's a good culture for discussing professional issues and talking about such things, and it indirectly impacts patient safety.”
**Subtheme 2c:** Continuity and cohesion in the team (*n* = 17)	Operating room nurse (ID09): “You know the people you work with, and you're aware of the qualities each one possesses, and communication flows very smoothly. It's not necessarily everything that's said, but there's just this understanding that you know, it's a bit like ((gestures with hands and eyes)) now I'm showing that we see each other and um (.) that we know what the other can do and what they're doing. The patient also notices, without them saying it, you can feel that the patient senses it when the team is coordinated, and things are going as they should.” Surgeon (ID13): “And it’s always nice if you are well familiar with a system and accustomed to working in a place where you know the people you work with, and you work in a team that you trust and have knowledge of their expertise.” Surgeon (ID17): “I know many personnel who work here regularly. But there are turnovers, so there may be personnel I don’t know. However, it’s very nice to know each other’s strengths and communicate or get along. Here, I know that the screw is quality assured in a way, or it’s that type of screw. In another place, when you don’t know them [colleagues], you might think you must double-check to ensure it is correct.”
**Theme 3. Proactive structures and participation in organizational learning**	
**Subtheme 3a:** Forum for discussing risk, safety, and events among allied healthcare professionals (*n* = 17)	Operating room nurse (ID12): “At one place, events were discussed at staff meetings approximately once a month. Then, they addressed the recurring incidents because they often have common themes. They discussed what we could improve to prevent it from happening again, and everyone participated and listened. They inquired if any actions had been initiated or if there were any suggestions on what actions to take to improve the performance. That's what I think the purpose of such a system is.” Surgeon (ID13): “The fact that the patient receives the best treatment with a predictable outcome is for the benefit of everyone involved: As an employee, colleague, and team member. It provides economically favorable results. So, it’s essential, and I wish that patient safety and quality work had a higher standing than randomized controlled studies at the receptor level.” Anesthesiologist (ID14): “The curse of working in a healthcare system is that when criticism arises, we often tend to close ranks within our profession. It means that when criticism arises, anesthesia professionals, for example, defend themselves against external attacks. Operating room nurses do the same if they receive criticism from anesthesia professionals. Surgeons do it if they receive criticism. I perceive this as almost uniformly negative, the fact that as we retreat into our professional, we miss the opportunities for improvement.”
**Subtheme 3b:** An efficient and formal system for reporting and learning from adverse events (*n* = 16)	Nurse anesthetists (ID04): “I often sense that physicians have an attitude that the most constructive approach is to talk about the event, thinking ‘we can just inform them’, with reporting seen as a means of holding someone accountable. Some of them seem to find it tidier to make a phone call. However, that`s not the essence of it. Reporting is not primarily about personal issues. It`s more often a systemic problem, a system issue.” Anesthesiologist (ID10): “I think the most important thing is communicating with the employees and ensuring functional reporting systems. I think that's the most crucial thing. If you don’t know, nothing will happen. But when you have the report, you must talk to the frontline workers to gather input and insight into the daily work. And what interventions are effective.” Surgeon (ID13): “It’s something I’ve requested and wanted more focus on. Because it’s obvious that events occur, and it’s something we can learn from. An event isn’t necessarily about the person who caused it. It’s more about the system, and we all need to learn from it, so we don’t only learn when we experience such an event ourselves.”

### Theme 1: Individual accountability as a safety net

With their competence and genuine ambitions for quality in patient treatment, the healthcare professionals experienced that they constituted a safety net for the patient in the surgical context. The individuals within the surgical team emphasized being well prepared, obtaining patient information, and having an overview of the situation. The team members strived to identify and prevent the risk of adverse events and to reduce the extent of injuries.

#### Subtheme 1a: Role understanding and interprofessional accountability

Surgical team members emphasized the importance of experience and an in-depth understanding of their and others’ roles in the operating room. This insight was considered crucial to ensure that the omission and commission actions were conducted correctly and at the right time. Additionally, a comprehensive overview enabled team members to contribute to vigilance, availability, and responsibility beyond their expertise. Experience in the operating room was also underscored as essential for building situational awareness, and team members should know when and how to respond and communicate during the surgical procedure.

#### Subtheme 1b: Competence and commitment to healthcare delivery

Competence, professional involvement, and patient engagement were shared values among surgical team members. They expressed their dedication and loyalty to their profession and aimed for effective and high-quality patient treatment by meticulously preparing and verifying the available information to prevent adverse events. Team members felt ethically obliged to provide respect, information, and person-centered care. Parallel to this, physicians conveyed personal ambitions and placed high prestige on their healthcare delivery and careers. This prestige culture led to work beyond regular working hours and a risk of a non-complaining culture, where the physicians had to prove their dedication and capability. The high prestige was considered positive for the patient’s treatment. However, it could also increase the workload and emotional demand, thus increasing hazards and risks. The physicians expressed that when an event occurred, the prestigious culture could increase feelings of shame and blame. Concerns about one’s career could make it appealing to keep events secret, increasing the emotional stress and the risk of encountering further incidents.

#### Subtheme 1c: Balancing work demands and patient safety on a knife edge

Surgical team members noted that efficiency demands threatened patient safety. Heavy workload, complex patient cases, lack of time for preparation, and severe consequences of making errors involved moral distress and exhaustion. Additionally, a culture of not speaking up regarding workload, especially among physicians, could affect patient safety and personal health. Experience and robustness were required to demand time to investigate ambiguous situations further. Anesthesia professionals were often worried about their workload and having too few resources for readiness. The heavy workload entailed challenges in training new colleagues, and such training contributed to an even higher workload. For nurses, being measured for effectiveness rather than professionalism could lead to demotivation and thoughts of leaving the profession. Experienced physicians indicated that they were less worried about the workload. However, they sought policy guidelines for treatments hospitals should and should not offer. Additionally, the physicians highlighted that the frontline professionals’ autonomy in patient treatment, involvement in administrative processes, and reduced clerical burdens were required to solve these challenges. They experienced that the workload threatened continuity in patient treatment and information.

### Theme 2: Psychological safety as a catalyst for well-being and safe performance in the operating room

A teamwork climate based on respect, cohesion, and psychological safety was considered essential for the surgical patient’s safety. Performed leadership and communication style influenced each team member’s performance and the team’s total achievement. Psychological safety and a culture of openness were encouraged to promote patients’ and healthcare professionals’ safety and to learn from events.

#### Subtheme 2a: Leadership and empowering communication

The participants had experienced various ways to lead and communicate in a surgical team. The surgeon was regarded as the leader and could lead and communicate in an empathic and empowering manner or a more authoritarian style with condescending communication. Participants from all specialties had experienced the negative effect of condescending communication. They described that such communication decreased the quality of their performance. The participants perceived that hierarchical and authoritarian leadership still existed in the operating room. However, the common perception was that empathic and egalitarian values gradually replaced this leadership style. Assertive leadership and affirmative communication increased the team members’ psychological safety, involvement, and performance quality. Team members were expected to be situationally aware of tense moments and condescending communication to empower the teamwork climate. Operating room nurses should ensure communication with the anesthetic professionals when the surgeons are in challenging situations. Inexperienced personnel found it more difficult to speak up, and experienced nurses built trust and respect by speaking up. The leading surgeon should encourage team members to speak up and emphasize their accountability.

#### Subtheme 2b: A supportive parachute and a culture of openness

The surgical team members understood that colleagues and managers would support them if an adverse event occurred. Nurses emphasized informal openness and individual learning from one another’s experiences. Physicians tended to seek support from a smaller number of allies they trusted and described previous experiences of cultures where events should be kept hidden due to hierarchy and prestige. Formal and informal role models provided essential guidance to create and maintain a supportive culture of openness. Managers and skilled, informal role models should share their adverse event experiences, promote the idea that everyone may experience an adverse event, and support colleagues who experience such an event. Managers and experienced role models should share their adverse event experiences to underscore that anyone can experience severe adverse events. The healthcare professionals’ demanding accountability and high levels of risk required a work environment that supported their psychological safety. They experienced a need for an immediate debrief after an adverse event, followed by a more systematic one. Managers should provide leaders or peer support for the involved parties. The surgical team members suggested that a culture without openness and support could result in healthcare professionals not processing their feelings after adverse events; They might become anxious, hurting their performance and threatening patient safety.

#### Subtheme 2c: Continuity and cohesion in the team

Team continuity and cohesion were accentuated to cultivate a teamwork climate that promoted patient safety, believed to be perceptible to the patients themselves, according to the healthcare professionals. They perceived that having trusted relationships with colleagues and knowing their competence contributed to a positive working environment and workflow, which could help maintain mental and physical balance during a surgical procedure. The surgical safety checklist was valuable for establishing a focused team with a shared understanding of the procedure. An extra ‘time out’ was considered effective to re-establish overview and communication if a situation turned chaotic and complex. Continuity during surgical procedures was pointed out as essential for the flow of information. Rapid changes in healthcare professionals, explicit use of substitute staff, and shift changes were considered potential threats to patient safety due to the lack of information transfer. The continuity of experienced personnel, few disruptions, and adequate staffing in the surgical department were perceived as facilitators for a calm and organized teamwork climate. Working environment and team cohesion were most mentioned and essential for nurses because the operating room is their primary work location.

### Theme 3: The importance of proactive structures and participation in organizational learning

The healthcare professionals identified a need for improved structural systems for reporting and learning from adverse events, near-miss incidents, and successful performance. They desired that hospital management emphasized the prioritization of patient safety and the statement that management and frontline personnel had a common goal: quality in healthcare delivery.

#### Subtheme 3a: Forum for discussing risk, safety, and events among allied healthcare professionals

Few forums existed for discussing patient safety and risk, especially interprofessional ones. Interprofessional forums for discussing operation plans, reviewing adverse events, and establishing a common understanding of safety hazards and prevention were pointed out to contribute to organizational learning and practical procedure improvements. The participants experienced that the information about reported adverse events was random and that patient safety should instead be on the agenda daily, weekly, and periodically, both within and between specialties. They wanted near-miss incidents and successful narratives to be shared along with adverse events in these forums. A need to reduce the barriers to discussing adverse events with staff from other specialties was identified. Additionally, the frontline experienced a gap between the patient safety work performed by administrators and frontline staff and wanted managers at different levels to establish physical contact and dialogue with frontline staff to focus on patient safety, a good work environment, and increased staff motivation. The participants highlighted that synchronizing the voices of frontline staff with academic and administrative work and decisions could improve surgical care.

#### Subtheme 3b: An efficient and formal system for reporting and learning from adverse events

The members of the surgical teams had varied attitudes toward and trust in the existing reporting systems. Nurses felt obliged to report events and emphasized the learning perspective of adverse events. However, they perceived the reporting process as time-consuming and needed more feedback about how the reports were handled and their consequences. The lack of feedback demotivated nurses from writing new adverse event reports, which made them worried about reduced insights and learning from adverse events. Physicians preferred to talk with involved parties after an event and avoid criticism of themselves or colleagues. However, they sometimes recorded the events in patient journals and emphasized the importance of explaining adverse events to patients. A need for a trusted, well-defined, open, and time-effective reporting system to facilitate improvement was identified. Frontline staff requested insight into administrative handling, involvement in event analysis, and discussions on pertinent improvement efforts. In addition, they thought that clinical registers and staff surveys could be used more systematically for organizational development and learning.

## Discussion

The results illustrated that patient safety culture in the surgical context needed to be addressed in processes and structures at multiple levels. At the individual level, this study’s participants emphasized ethical commitment and high-quality patient-centered care. In clinical processes at the team level, the teamwork climate was crucial for patient safety, healthcare professionals’ well-being, and organizational learning. In addition, the participants pointed to structural factors influencing the patient safety culture. This complexity aligned with Donabedian’s model for assessing healthcare quality and previous studies reporting that structural interventions, such as the safe surgery checklist, improve care processes and patient outcomes [28–30].

This study highlighted psychological safety in clinical processes as crucial for patient safety in the operating room. Psychological safety is characterized by a trusting and respectful collaboration in which team members feel free to speak up without interpersonal risk [31]. To feel free to speak up and challenge authorities is connected to safety culture [32]. Building and sustaining trust may be difficult, especially in hierarchical healthcare teams [6]. Participants in this study described how assertive leadership styles and empowering communication methods made them engage rather than withdraw from situations. These non-technical skills are considered essential for safety culture and are included in the ‘formula for survival in surgery’ for improved quality [33]. Leadership influences psychological safety, hence the team’s total involvement and performance [19, 34]. Encouraging and involving the whole team to share ideas and ask questions about patient safety, in and between specialties, can prevent adverse events, reduce costs, and increase patient satisfaction [32, 35, 36]. This study added that each team member must acknowledge their responsibility to speak up and build a trusting working relationship. Education, experience, and knowledge are individual characteristics that increase the confidence to speak up [37]. The results underlined the importance of retaining experienced team members, minimizing temporary replacements, and providing inexperienced team members with education and supervision. Simulation and communication training could improve the teamwork climate [32, 38, 39]. However, feeling confident to speak up begins with team familiarity, supportive leadership, and having a shared understanding of safety, checklists, and transparency regarding events [32, 35, 37, 38, 40].

Psychological safety enables a culture of openness and organizational learning [34]. The results of this study emphasized the importance of informal role models in creating communication openness and learning, especially in prestigious cultures. Supervisors and experienced personnel may contribute by fostering the acceptance of events, sharing their stories, and developing an environment conducive to learning from adverse events [34]. The participants described the feeling of shame and blame as inescapable emotions due to the commitment to maintain the patient`s safety and personal prestige. The literature supports this result [17, 41–43], and experiencing adverse events is associated with symptoms of emotional exhaustion, burnout, and turnover intentions [17]. Burnout among surgical residents is described as an epidemic, and the stress in the surgical context is described as constant [35]. This knowledge clarifies that support when an adverse event occurs is essential to prevent negative emotions such as anxiety and fear of future adverse events that influence cognitive functions [17]. This study added weight to the suggestion that healthcare professionals’ well-being may affect patient safety [38, 44, 45]. A previous review and this study highlighted the absence of structured peer support and debriefing after an adverse event [43]. These organizational processes may enable healthcare professionals to remain in their profession without experiencing intractable fear [16, 17, 41, 42].

This study highlighted the need for interprofessional arenas where the surgical team could work on a shared understanding of patient and psychological safety and learn from adverse events. O’Donovan [38] supports this need, claiming that learning across specialties is essential in complex collaborations, such as a surgical team. A recent review of ‘Morbidity and Mortality Meetings’ reports that well-implemented and administered meetings are related to organizational learning and patient safety culture facilitates the effect of these meetings [46]. Additionally, this latter review points to the need for clinical engagement in quality improvement [46]. O’Donovan [38] supports the need for arenas where hospital leadership meets the frontline workers to understand their perspectives on the quality of care. The lack of these organizational processes and structural factors, such as a heavy workload and administrative burden, could threaten patient safety [35]. This raised some suggestions for improvement in the surgical context. Quality improvement should be grounded in the current context and considered according to other processual and structural variables [38]. The ‘human errors’ described in the literature [14, 15] should be analyzed with frontline workers and include structural perspectives, such as staff change, workload, time pressure, ergonomics, and equipment [47]. Likewise, there could be justifications for exploring patients’ perceptions and experiences regarding adverse events. Finally, leaders should promote proactive learning from successful pathways for a positive learning perspective because of the high number of successful surgical procedures [38, 44, 48]. Focusing on success and learning from excellence represent a new paradigm of safety, described as the ‘safety 2-perspective’ and ‘resilience engineering’ in the literature [48–50]. ‘Safety 2’ represents approaches and methodologies to learn from success, and resilience refers to the robustness and the ability to adjust the healthcare performance due to variations in the actual conditions. Thus, understanding and increasing resilience at multiple organizational levels should complement the traditional ‘safety 1-perspective’ focusing on understanding adverse events retrospectively to prevent errors [49].

This study had multiple strengths. First, there was a high information power in the data. Second, the shared ideas and common patterns of meaning generated from a heterogeneous dataset were valuable [22]. Third, methodological integrity and rigorous analysis increased trustworthiness. Audit trails, author analysis meetings, and presented quotations ensured credibility, confirmability, and authenticity. The descriptive data of the participants increased the dependability and the reader’s possibility to evaluate the relevance in other settings. However, this study also had limitations. First, safety cultural perspectives were contextually bonded and limited to general and orthopedic surgeries in two large, urban Norwegian hospitals. Second, the reflexivity in the analytical process may have influenced the authors’ interpretations. Finally, the participants shared what they felt like, which could constitute self-selection bias and influence the potential to capture the intangible dimensions.

## Conclusion

This explorative study contributed to increased knowledge of patient safety culture in a surgical context by highlighting the importance of healthcare professionals’ experience and competence, psychological safety in surgical teams, and structural systems promoting organizational learning. Further research might include processual interventions for creating a culture of psychological safety and openness and structural improvements as a trusted reporting system, interprofessional forums, and forums for frontline personnel and the administration to discuss patient safety. Finally, the perspectives of the surgical team could be complemented by surgical patients’ perspectives.

### Supplementary Information

**Additional file 1.** Mind Map.

**Additional file 2.** Interview Guide.

**Additional file 3.** Reflexive Thematic Analysis.

## Data Availability

No datasets were generated or analysed during the current study.
