# Supplementary material for: The anatomy of safe surgical teams: an interview-based qualitative study among members of surgical teams at tertiary referral hospitals in Norway
Source: Patient Saf Surg. 2024 Feb 19;18:7. doi: 10.1186/s13037-024-00389-w (PMC10877820; doi:10.1186/s13037-024-00389-w)
Supplement: Supplementary file 1 — Additional file 1. Mind Map. [file 13037_2024_389_MOESM1_ESM.docx]

**Project: Patient safety culture and adverse events**

**Mind map:** We want you to create a mind map by writing or drawing to help you remember and reflect on your experiences and perceptions. You can choose to use the template below or make a visual representation. Please avoid putting your name on the mind map. Alternatively, you can choose to write about an event. One suggestion is to create the mind map or text, set it aside for a while, and revisit it later.

What are the objectives of the mind map? The main aim of the mind map is to enhance your reflections. Second, if you consent, we would appreciate receiving your mind map, anonymising it to ensure it cannot be traced back to you, and including it in our analysis. Alternatively, it may be used as a visual representation in a published article. If you do not want to share the mind map, you can dispose of it, or we can.
