# Supplementary material for: The anatomy of safe surgical teams: an interview-based qualitative study among members of surgical teams at tertiary referral hospitals in Norway
Source: Patient Saf Surg. 2024 Feb 19;18:7. doi: 10.1186/s13037-024-00389-w (PMC10877820; doi:10.1186/s13037-024-00389-w)
Supplement: Supplementary file 2 — Additional file 2. Interview Guide. [file 13037_2024_389_MOESM2_ESM.docx]

**Interview guide for members of the surgical teams**

1. First, I would like us to talk about the safety of the surgical patient in the operating room. Can you describe a positive experience where the patient’s pathway went as you want? Possible follow-up questions:

- What are your experiences regarding how the leadership closest to clinicians works to reduce the risk of adverse events in the operating department? What are your general thoughts on this?
- How do you perceive the efforts to ensure the safety of surgical patients across departments?
- Can you tell me how you work to reduce the risk of patient harm within the operating room?
- Can you recount a situation where communication and collaboration influenced the risk of an adverse event in patient care? And vice versa.

1. Can you share an adverse event you have experienced in, or related to, the operating room? Possible follow-up questions:

- What do you think influenced the risk in that situation?
- How do you perceive how management and colleagues handled the situation?
- How did you and those involved in this event experience it?
- How do you perceive the communication surrounding adverse events and quality improvement in the department?

1. It is expected to experience near-miss incidents that could have harmed the surgical patient. What are your experiences with this? Possible follow-up questions:

- What do you think contributes to such near-miss incidents, and how is harm prevented in such situations?
- How do you perceive your department handling such near-miss incidents?
- What do you think is required of you and each physician/nurse to reduce the risk of adverse events in daily work?
- How do you think the department as a collective can contribute to increased patient safety? And leadership/ management above the departmental level?

In conclusion, is there anything else you would like to add, something I haven`t asked about?

Summarise/clarify.
