# Supplementary material for: The anatomy of safe surgical teams: an interview-based qualitative study among members of surgical teams at tertiary referral hospitals in Norway
Source: Patient Saf Surg. 2024 Feb 19;18:7. doi: 10.1186/s13037-024-00389-w (PMC10877820; doi:10.1186/s13037-024-00389-w)
Supplement: Supplementary file 3 — Additional file 3. Reflexive Thematic Analysis. [file 13037_2024_389_MOESM3_ESM.docx]

# Reflexive thematic analysis

| **Researcher’s reflexivity:**  MV (MD, PhD candidate, and operating room nurse ♀) conducted the interviews. This was her third qualitative interview study. M. was familiar with some of the participants from previous job situations but not in-depth. MV told the participants that her profession was operating room nursing. However, in this study, she worked as a researcher aiming to better understand patient safety culture and adverse events in the surgical context. MV also provided theoretical background and information about MV’s PhD project.  The first interview was a pilot interview; however, the data was included, and no revisions in the semi-structured interview guide were needed. | | | |
| --- | --- | --- | --- |
| **Phase** | **Name of phase** | **Period** | **How we conducted the phase** |
| 1 | **Familiarising yourself with the dataset**  Description:  Aim to become deeply familiar with the data material as cases and as a whole. | January-  May 2023 | MV listened to the audiotapes and revised the automatic Word transcript manually twice, according to Braun and Clarke’s method for verbatim transcription, and ensured the quality of the transcripts by listening and reading a third time. MV made brief notes about analytic ideas.  SOD listened to the audiotapes to ensure the quality of the dialogue and the transcripts and ensured that meaning was maintained in reworded unidentified paragraphs.  One participant read the respective transcript to ensure the content and to confess that meaning was maintained in the reworded unidentified paragraphs.  Finally, MV listened and wrote preliminary codes. |
| 2 | **Coding**  Description:  Identify segments relevant to the research question and apply meaningful code labels to segments. | May 2023 | MV coded systematically in NVivo 12 in Services for Sensitive Data (TSD). The first examination was inductive and data-driven and ended in 81 codes. Codes were segments of data representing a single idea. Before the second examination for coding, MV drew inspiration from existing theory and previous research to reflect on what the data could contribute to the literature. With this deductive inspiration, MV coded a second time. The coding was still manifest and close to the data, descriptive, and with a low degree of abstraction, conceptualisation, and interpretation. Some of the first codes were merged, some were divided, and some were reworded. The second examination in this phase ended in 56 codes. The codes were data-driven; in addition, they reflected the theoretical background.  MV and SOD discussed initial coding, the idea in each code, and relevant concepts. |
| 3 | **Generating initial themes**  Description:  Aim to identify patterns of shared meaning throughout an active and constructing process. | June 2023 | MV clustered similar codes with similar ideas to 11 “code clusters” that shared a core idea. MV generated 6 “candidate themes” based on the code clusters using patches, posters, and whiteboards. MV and SOD discussed the candidate themes with an inductive and deductive approach and finished 4 initial themes (17 subthemes) with a higher level of abstraction, interpretation, and conceptualisation. |
| 4 | **Developing and reviewing themes**  Description:  Aim to ensure that themes fit the dataset and each theme concerns a shared meaning and the essential meanings from the dataset. | June 2023 | MV reviewed the data material in a new NVivo 12 file to test how the data fit the initial themes and subthemes.  Subthemes were raised as themes, themes were reduced to subthemes, subthemes were merged, and concepts were changed as the dynamic, iterative process increased the understanding of latent content.  MV presented and discussed 4 themes and 15 subthemes with SOD, ECTD, and ASH. To engage the team with the data, MV presented the process of generating themes and subthemes with core characteristics, quotations, and key points related to the research questions.  MV reviewed the themes and subthemes according to the team’s inspiration and feedback, ensured the themes were reviewed with SOD, and went back to the data material. After the supervision and reviewing process, MV did minor revisions and reviewed the data material twice in NVivo 12. |
| 5 | **Refining, defining, and naming themes**  Description:  Aim to refine the themes and ensure they are demarcated with a strong core concept and a concise name. | July- August 2023 | MV presented 3 refined and renamed themes with a brief synopsis and 8 subthemes with the essence, core concepts, and quotations for SOD, ECTD, and ASH. It was agreed that the themes and subthemes reflected the data material and were abstracted to an appropriate level. The team discussed how the themes constitute an overall story, how the results contribute to the existing theory, and what to emphasise in the discussion. At this point, the team agreed that there was no overarching theme.  MV sent documents from the analysis process to the rest of the project group members: BTV, AKB, and VSH. |
| 6 | **Writing up**  Description:  Aim to weave the results into a coherent story and answer the research questions. | September-  November 2023 | MV wrote the results and the draft of the article, and all the project members contributed to the writing process. In this phase, MV read through the field notes and memos to memorise reflections throughout the data collection and analysis.  The draft was sent to the project group, and the co-authors contributed to the manuscript.  All the co-authors gave their final approval to the final submitted manuscript. |

**Example analytical process:**

| **Codes–**  **example of multiple codes per code cluster** | **Code Clusters/ Initial Subthemes**  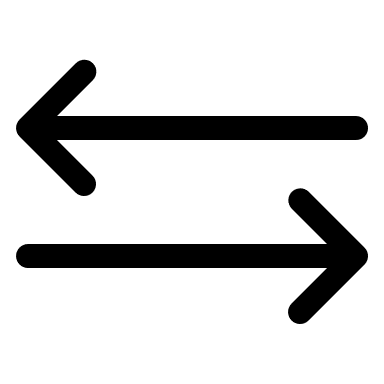 | **Initial Themes**  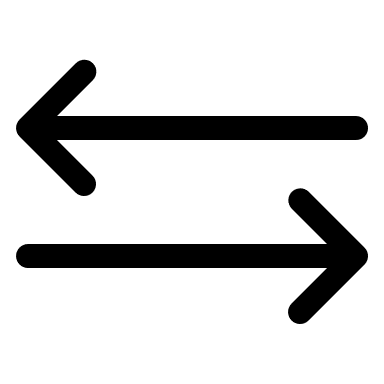 | **Subthemes**  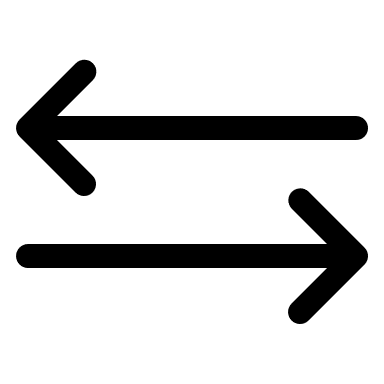 | **Themes** |
| --- | --- | --- | --- | --- |
| Know your and other team members’ function | Role understanding and overview | Situation awareness | Role understanding and interprofessional accountability | **Individual accountability as a safety net** |
| Know when you can talk and not | Situation-based communication |  |  |  |
| Time for training and supervision is important | Training and the feeling of coping |  | Competence and commitment to healthcare delivery* |  |
| Experienced personnel are valuable for patient safety | Experience and resilience |  | Balancing work demand and patient safety on a knife edge** |  |
| Dedication to healthcare delivery | Competence and prestige | Professional ambition and commitment | *Competence and commitment to healthcare delivery |  |
| The workload threatens patient safety | Balance workload, effectiveness, and quality |  | **Balancing work demand and patient safety on a knife edge |  |
| Time to read and obtain information about patient safety | Time to be prepared |  | *Competence and commitment to healthcare delivery |  |
| To feel shame and blame is inevitable | Shame and blame when an event occurs |  | *Competence and commitment to healthcare delivery |  |
| A changing hierarchy | Hierarchy and leadership | Team climate and psychological safety | Leadership and empowering communication | **Psychological safety as a catalyst for well-being and safe performance in the operating room** |
| Condescending communication reduces the healthcare professionals’ safety | Safety and involvement |  | A supportive parachute and a culture of openness*** |  |
| To know each other increases the feeling of a safe pathway | Continuity and care of colleagues |  | Continuity and cohesion in the team |  |
| “Safe surgery” makes the team focus on a common goal | Setting the team in a “time out” |  |  |  |
| Affirmative like “closed loop” communication increases workflow | Work climate and constructive communication |  |  |  |
| Formal and informal role models should share their experiences on events | Formal and informal role models*** | Culture for openness and learning | Forum for discussing risk, safety, and events among allied healthcare professionals | **The importance of proactive structures and participation in organisational learning** |
| We experience a lack of feedback about adverse events | Well-defined and effective system for improvement |  |  |  |
| We support and learn from each other’s experiences | Informal dialogue and support when an event occurs*** |  | An efficient and formal system for reporting and learning from adverse events |  |
| We do not have debriefs | Systematic in handling when an event occurs |  |  |  |
